# Chitosan against cutaneous pathogens

**DOI:** 10.1186/2191-0855-3-37

**Published:** 2013-07-06

**Authors:** Jackson Champer, Julie Patel, Nathalie Fernando, Elaheh Salehi, Victoria Wong, Jenny Kim

**Affiliations:** 1 Division of Dermatology, David Geffen School of Medicine at UCLA, Los Angeles, CA 90095, USA; 2 Division of Immunology, Beckman Research Institute, City of Hope National Medical Center, 1500 E. Duarte Road, Duarte, CA 91010, USA; 3 Department of Dermatology, Greater Los Angeles Healthcare System Veterans Affairs, Los Angeles, CA 90073, USA

**Keywords:** Chitosan, Benzoyl peroxide, Acne vulgaris, *Propionibacterium acnes*, *Staphylococcus aureus*, Antibacterial

## Abstract

*Propionibacterium acnes* and *Staphylococcus aureus* are cutaneous pathogens that have become increasingly resistant to antibiotics. We sought to determine if chitosan, a polymer of deacetylated chitin, could be used as a potential treatment against these bacteria. We found that higher molecular weight chitosan had superior antimicrobial properties compared to lower molecular weights, and that this activity occurred in a pH dependent manner. Electron and fluorescence microscopy revealed that chitosan forms aggregates and binds to the surface of bacteria, causing shrinkage of the bacterial membrane from the cell wall. Of special relevance, clinical isolates of *P. acnes* were vulnerable to chitosan, which could be combined with benzoyl peroxide for additive antibacterial effect. Chitosan also demonstrated significantly less cytotoxicity to monocytes than benzoyl peroxide. Overall, chitosan demonstrates many promising qualities for treatment of cutaneous pathogens.

## Introduction

Chitosan is derived from the partial deacetylation of chitin, a natural polysaccharide composed of *β*1 → 4 linked N-acetylglucosamine. It has demonstrated potential as a vehicle for drug and DNA delivery via nanoparticles (Singla and Chawla 2001), as a food preserving agent (No et al 2007), and as a wound dressing for severely hemorrhaging injuries (Brown et al. 2009). Chitosan is also known to have antimicrobial activity against viruses (Kurita 2006), fungi, and bacteria (Rabea et al. 2003), which combined with its high biocompatibility, low toxicity, and ability to biodegrade, make it a promising candidate for medical use against various pathogens (Kong et al. 2010). Chitosan has already been shown to be effective *in vivo* against bacteria (Lee et al. 2009; Moon et al. 2007).

Two common cutaneous pathogens, *Propionibacterium acnes* and *Staphylococcus aureus*, have increasingly developed resistance to frontline antibiotics (Song et al. 2011; Patel et al. 2010; Jappe et al. 2008), necessitating the development of alternative treatments. Benzoyl peroxide is a common treatment for acne vulgaris, but it is highly toxic to human cells, making it less than ideal (Kraft and Freiman 2011). In addition, benzoyl peroxide is very irritating, resulting in poor patient compliance. Topical retinoids are also irritating, and oral isotretinoin, while extremely effective for severe acne, has significant adverse effects, with restrictions on its use implemented by the FDA. Therefore, there is a need to develop novel therapeutics that can effectively treat infectious dermatological diseases without harmful side effects.

Thus, we sought to determine if chitosan has the potential to act as an effective antibacterial agent against *S. aureus* and *P. acnes*, with a focus on treatment of the latter, which has been identified as a factor in the pathogenesis of acne vulgaris (Bellew et al. 2011; Kurokawa et al. 2009). Additionally, we sought to determine the parameters to optimize chitosan’s antibacterial properties and investigated the antibacterial mechanism of chitosan, which is not fully understood.

## Methods

### Materials

Chitosan with 75%+ deacetylation and varying molecular weight was obtained from Aldrich. Chitosan was added at 1 mg/mL to 0.01N aqueous HCl, allowed to dissolve, and then adjusted with NaOH to pH 6. Unless otherwise specified, experiments were performed with high molecular weight chitosan (310-375+ kDa).

Benzoyl peroxide was obtained from Sigma and dissolved in DMSO at 10 mg/mL, immediately diluted to appropriate concentrations, and added to treatments. Final DMSO concentration was 1%, a concentration found not to affect results.

8 strains of *P. acnes* clinical isolates were obtained from the faces of human volunteers using the tape-strip method, a protocol approved by the Institutional Review Board at UCLA.

### *P. acnes* colony-forming unit assay

*P. acnes* American Type Cell Culture (ATCC) strain 6919 were grown anaerobically at 37°C in Reinforced Clostridial Media (Oxoid) for 3 days and collected in the exponential phase of growth by centrifugation. Bacteria were washed with pH 6 sodium phosphate buffer supplemented with 0.03% Trypticase Soy Media and quantified by reading with a spectrophotometer at 600 nm and applying a conversion of ~10^8^ CFU = 1 absorbance unit. Bacteria were then added to treatments at 10^7^ CFU/mL in a volume of 400 μL and incubated aerobically at 37°C for 3 hours. Control samples were untreated, and the aerobic incubation was found to have no effect on bacteria viability. Dilutions were conducted, and each sample was plated on brucella agar with 5% sheep blood supplemented with hemin and vitamin K (Remel). Plates were incubated anaerobically at 37°C for 3 days, allowing visible *P. acnes* colonies to form, and colonies were counted to determine concentration before plating.

### *S. aureus* colony-forming unit assay

*S. aureus* strain SH1000 were grown overnight at 37°C in Trypticase Soy Broth (Becton-Dickinson), then diluted 1:50 into fresh media, and collected 3 hours later in the exponential phase of growth by centrifugation. Bacteria were washed with pH 6 sodium phosphate buffer supplemented with 0.03% Trypticase Soy Media and quantified by reading with a spectrophotometer at 600 nm and applying a conversion of ~5 × 10^8^ CFU = 1 absorbance unit. Bacteria were then added to treatments at 10^7^ CFU/mL in a volume of 400 μL and incubated aerobically at 37°C for 3 hours. Control samples were untreated. Dilutions were conducted, and each sample was plated on Trypticase Soy Agar. Plates were incubated at 37°C overnight, allowing visible *S. aureus* colonies to form, and colonies were counted to determine concentration before plating.

### Electron microscopy

*P. acnes* were grown as described above, but treatments contained 1mL volume with 250 μg/mL chitosan at a concentration of 10^8^ bacteria/mL. Bacteria were incubated aerobically for one hour at 37°C, washed 3x with PBS solution, and resuspended in PBS with 2% glutaraldehyde. The samples were fixed with 0.05% osmium tetroxide for 5 minutes, dehydrated in graded ethanol, and embedded in Eponate 12 (Ted Pella). 60-70 nm sections were cut on a Reichert-Jung Ultracut E ultramicrotome and picked up on formvar coated copper grids. The sections were stained with uranyl acetate and Reynolds lead citrate, and examined on a JEOL 100CX electron microscope at 80kV.

### Fluorescence microscopy

Chitosan was labelled with FITC as conducted previously (Qaqish and Amiji 1999). Chitosan was dissolved at 5 mg/mL in 0.1M acetic acid. An equal volume of methanol with 0.25 mg/mL FITC was slowly added while stirring. The solution was kept in the dark for one hour, and then chitosan was precipitated with NaOH. The chitosan pellet was collected by centrifugation, washed 3x with water, dissolved at 1 mg/mL in 0.01N HCl, and adjusted with NaOH to pH 6. *P. acnes* was grown and washed as described above, except that 100 μL of 50 mM CTC solution was added to 3mL bacteria cultures before harvesting. 100 μg/mL FITC-labeled chitosan was added to ~10^7^ bacteria/mL, and viewed one hour later with a Leica Microsystems TCS-SP2-AOBS Confocal Microscope. An argon 488 nm laser was used for excitation. Green FITC was viewed through a 500-535 nm wavelength filter, and red CTC was viewed through a 599-700 nm wavelength filter.

### MTS assay

Blood was drawn from healthy human volunteers according to a protocol approved by the Institutional Review Board at UCLA. PBMCs were isolated by Ficoll-Paque (Pharmacia) gradient and allowed to adhere for 2 hours in RPMI media with 1% Fetal Calf Serum (Omega Scientific) in 96-well plates. Cells were washed 3x with RPMI media to obtain adherent monocytes. The HaCaT cell line of keratinocytes were cultured. Cells of both types were incubated at 37°C with treatment in 100 μL RPMI media with 10% Fetal Calf Serum (monocytes) or HaCaT media (keratinocytes) for 16 hours. 20 μL MTS assay reagent (Promega) was added to each well, and cells were allowed to incubate for 4 hours at 37°C. The absorbance of each well was read at 490 nm, with absorbance proportional to the number of viable cells (Cory et al. 1991).

## Results

### Chitosan demonstrates antimicrobial effect against *P. acnes* and *S. aureus*

**Figure 1:**
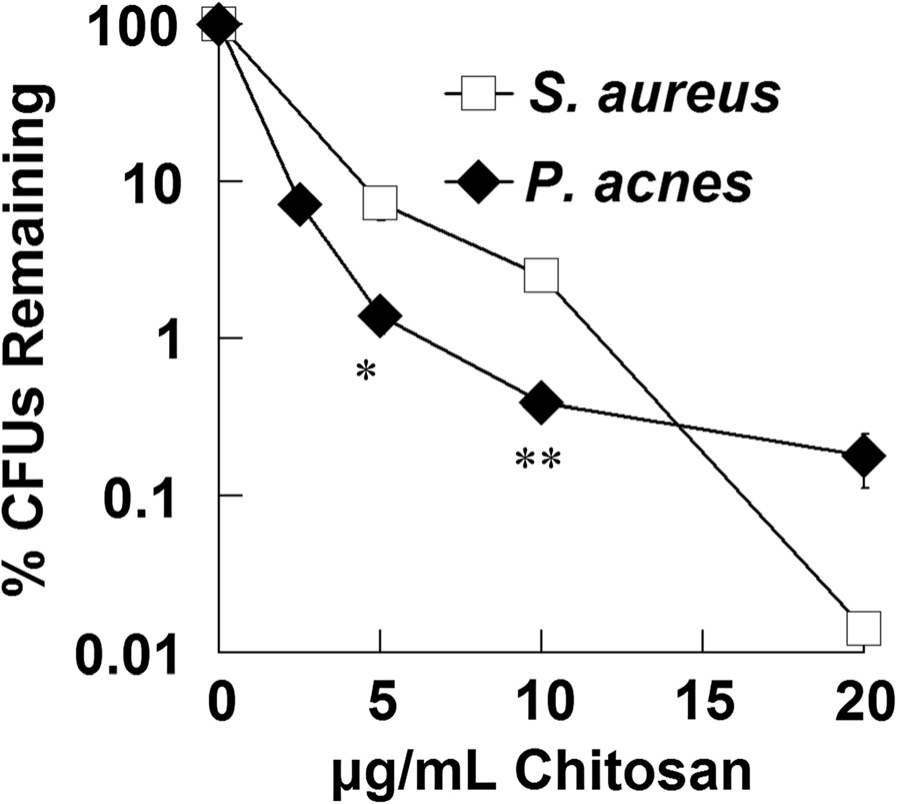
**Chitosan demonstrates antimicrobial effect against *P. acnes *and *S. aureus*.** Chitosan was incubated with bacteria for 3 hours to assess antibacterial activity. Bacteria was then enumerated by colony-forming unit assay to determine concentration, which was plotted as a percentage of untreated sample. Error bars represent SEM (n = 4, independent experiments). *p < 0.05 and **p < 0.01 (t-test).

Chitosan demonstrated a concentration-dependent antimicrobial activity against both *P. acnes* and *S. aureus* (Figure 1). Chitosan at 5 μg/mL inhibited *P. acnes* growth by 2 logs and at 10 μg/mL by close to 3 logs. At these lower concentrations, chitosan was more effective at inhibiting *P. acnes* growth than *S. aureus*, which was inhibited by 1 log at 5 μg/ml and by 2 logs at 10 μg/mL. At a higher concentration of chitosan (20 μg/mL), chitosan was effective at inhibiting *S. aureus* growth with virtually no *S. aureus* remaining. This may be because *S. aureus* remained in exponential growth throughout the experiment, while *P. acnes*, an anaerobic species, began to shift to a static phase. It has been shown chitosan is most effective against bacteria in the exponential phase of growth (Chen et al. 2002).

### High molecular weight chitosan has greater antibacterial activity

**Figure 2:**
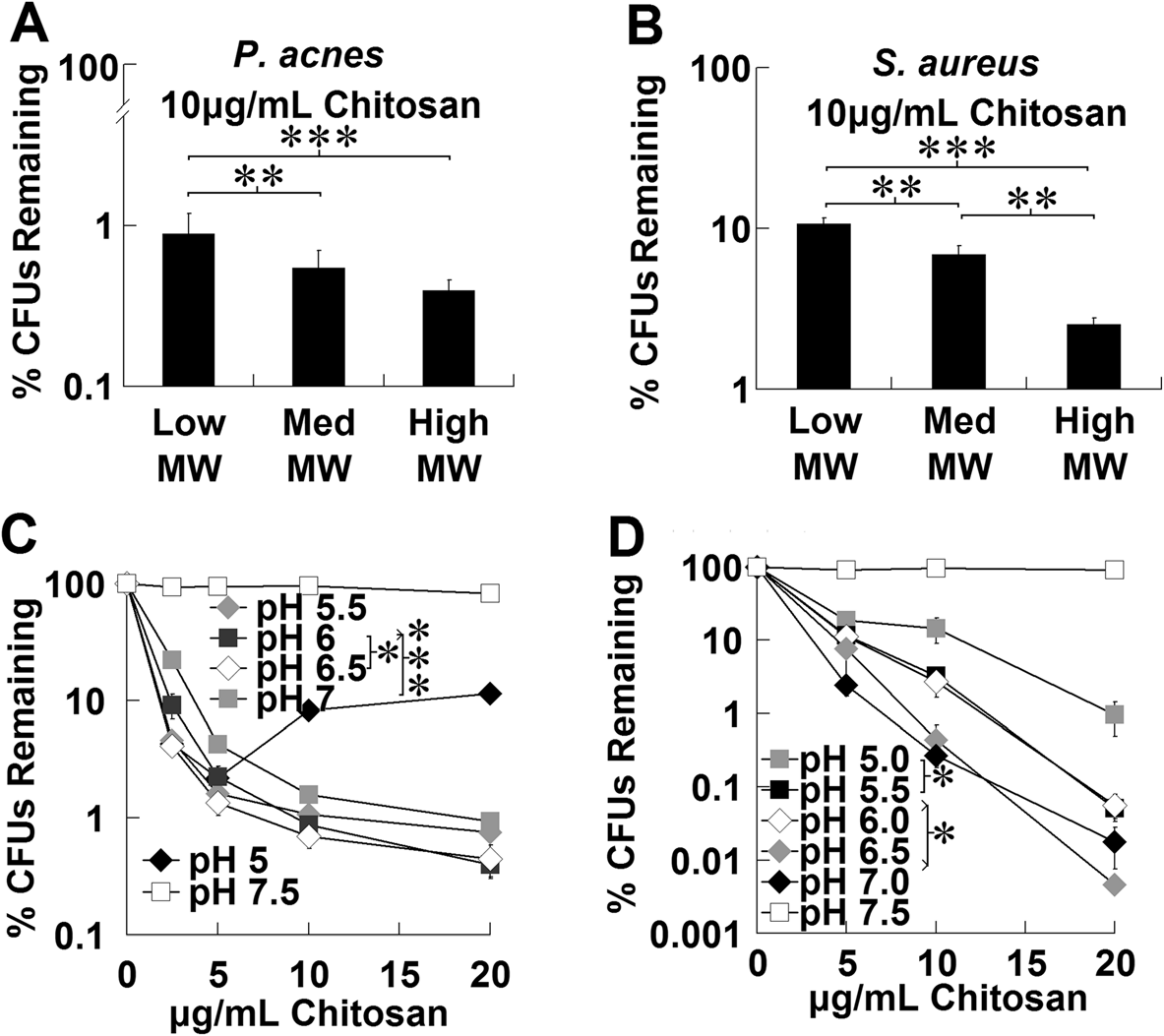
**Molecular weight and pH affect chitosan antibacterial activity. (A**, **B)** Chitosan of low (50-190 kD), medium (190-310 kDa), or high (310-375+ kDa) molecular weight or **(C**, **D)** chitosan at different pH was incubated with **(A**, **C)***P. acnes* or **(B**, **D)***S. aureus* for 3 hours to assess antibacterial activity. Bacteria was then enumerated by colony-forming unit assay to determine concentration, which was plotted as a percentage of untreated sample. Error bars represent SEM (n = 3 for **A**, **C**, **D**, n = 4 for **B**, independent experiments). *p < 0.05, **p < 0.01, and ***p < 0.001 **(**sign test across all concentrations for **A**, **C**, **D**, paired t-test for **B)**.

To determine the effect of molecular weight (MW) on antibacterial activity, chitosan of low MW (50-190 kDa), medium MW (190-310 kDa), and high MW (310-375+ kDa) were tested. Concentrations of 2.5, 5, 10, and 20 μg/mL were tested against *P. acnes* and 5, 10, and 20 μg/mL against *S. aureus*. Data at 10 μg/mL was typical of results for all concentrations (Figure 2A-B). Chitosan of high molecular weight had greater effect against the gram-positive bacteria *P. acnes* and *S. aureus*, with molecular weight having a more pronounced impact against *S. aureus*.

### Chitosan antibacterial activity is pH dependent

Chitosan was tested against *P. acnes* and *S. aureus* at various pH levels (Figure 2C-D). For *P. acnes*, which did not multiply during the experiment, chitosan was observed to be more effective at lower pHs, with pH 5.5-6.5 having indistinguishable activity. An anomalous hook effect was apparent in all trials at pH 5. Low pH had an antibacterial effect, but this was small compared to the effect of chitosan. Chitosan’s positively charged amine group has a pK_a_ of approximately 6.3, and thus, its solubility falls rapidly in this pH region. The stock chitosan solutions used had partially precipitated at pH 7, and fully precipitated at pH 7.5, when the amine group has minimal positive charge. We observed chitosan to be almost completely ineffective at pH 7.5. For *S. aureus* at lower pHs, however, we saw chitosan effectiveness generally decline with reduction in pH, which was correlated with a reduction in the bacterial growth rate for the duration of the experiment, as determined by untreated controls.

### Electron microscopy shows chitosan alters cell surface of *P. acnes*

**Figure 3:**
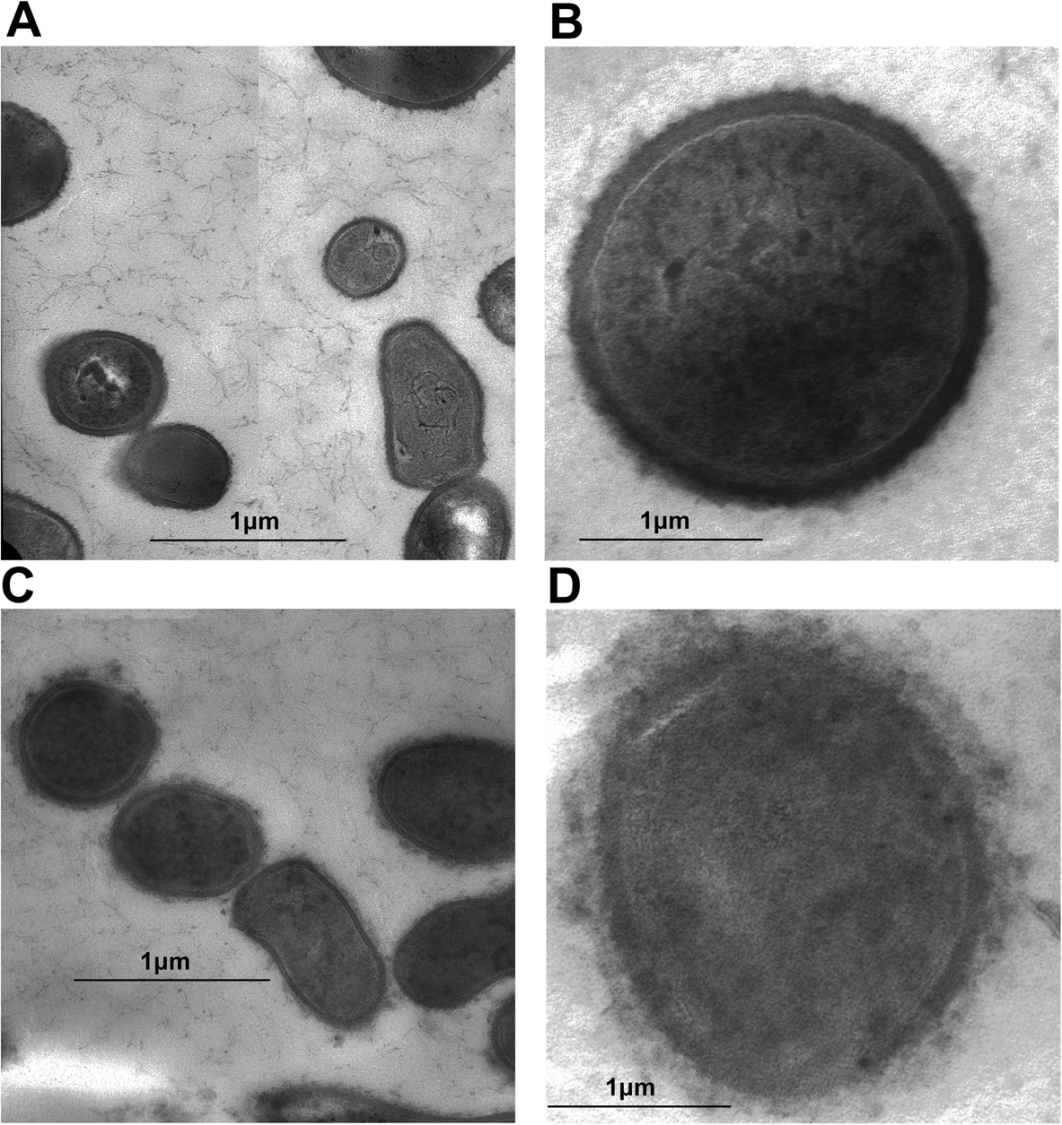
**Electron microscopy shows antibacterial effect. ***P. acnes* bacteria were **(A**, **B)** left untreated or **(C**, **D)** incubated for one hour with chitosan, and then prepared for electron microscopy. Images were taken at 19,000x magnification with 1 μm scale bar **(A**, **C)** or 72,000x magnification with 250 nm scale bar **(B**, **D)**. Figure 4A was modified by moving two parts of a larger image together. Images are representative of two separate experiments.

Chitosan is thought to exert its antibacterial activity by disturbing the integrity of the cell membrane. To determine if a similar mechanism is employed in the killing of *P. acnes*, we examined chitosan-treated bacteria using transmission electron microscopy. Micrographs of untreated *P. acnes* illustrate the bacterium's normal surface architecture, which appears smooth and sharply layered (Figure 3A-B). In contrast, the chitosan-treated bacteria lost the integrity of their surface structure, which appears disturbed, losing its crisp, well-defined structure (Figure 3C-D). Chitosan bound to the outside of the cell wall is readily apparent. In addition, we observed the *P. acnes* membrane to be shrunken, and out of contact with the cell wall in places. These images reveal that chitosan perturbs the surface integrity of *P. acnes*, which could account for its antimicrobial activity.

### Fluorescence confocal microscopy shows chitosan-bacteria interaction

**Figure 4:**
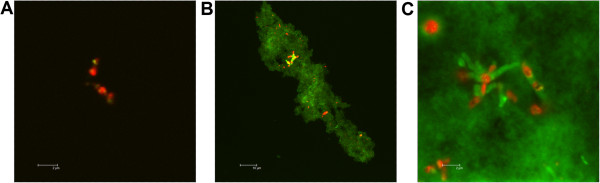
**Fluorescence confocal microscopy shows chitosan-bacteria interaction.** CTC-stained *P. acnes* bacteria were **(A)** left untreated or **(B**, **C)** incubated for one hour with FITC-labeled chitosan, and then examined by fluorescence microscopy. Scale bar is 2 μm **(A**, **C)** or 10 μm **(B)**. Bacteria is red, while chitosan is green.

Cyanoditolyl Tetrazolium Chloride (CTC)-stained *P. acnes* was treated with Fluorescein Isothiocyanate (FITC)-labeled chitosan and examined via fluorescence microscopy. Untreated bacteria were distributed evenly in solution (Figure 4A). However, when treated with chitosan, bacteria were clustered into small chitosan particles (Figure 4B) often visible to the naked eye. Chitosan particles ranged in size from 5-200 μm, and contained 0–50 individual bacteria. All observed bacteria were bound to these particles. Furthermore, within the particles of chitosan, *P. acnes* bacteria were often found in areas of particularly high chitosan concentration (Figure 4C). The chitosan appears directly attached to the surface of the bacteria, where it likely exerts its antimicrobial action.

### Clinical isolates of *P. acnes* are sensitive to chitosan

**Figure 5:**
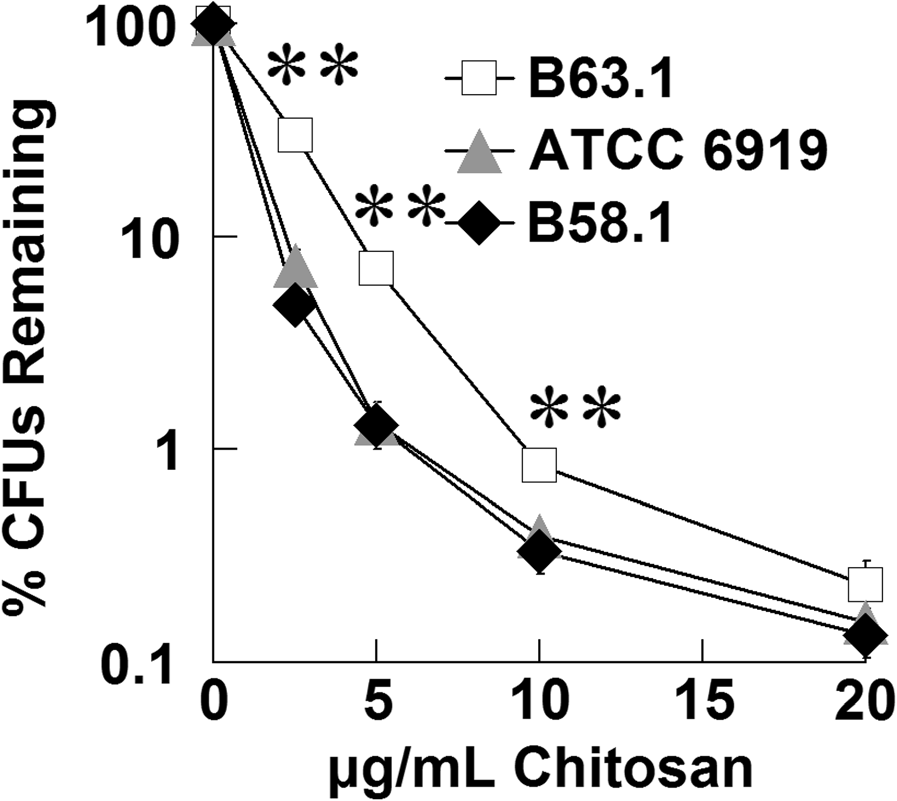
**Clinical isolates of *P. acnes *are sensitive to chitosan.** Chitosan was incubated with different strains of *P. acnes* for 3 hours to assess antibacterial activity. Bacteria was then enumerated by colony-forming unit assay to determine concentration, which was plotted as a percentage of untreated sample. 8 clinical isolates were tested, and the most and least susceptible to chitosan are displayed along with strain ATCC 6919. Error bars represent SEM (n = 3, independent experiments). **p < 0.01 (paired t-test).

To determine potential antimicrobial activity in clinical settings, we tested chitosan against *P. acnes* isolates from 8 different acne patients (Figure 5). All showed similar vulnerability to chitosan. One strain, B63.1, demonstrated somewhat more resistance compared to *P. acnes* ATCC strain 6919 at 5 and 10 μg/mL chitosan, but at 20 μg/mL, all strains were reduced by nearly 3 logs.

### Chitosan and benzoyl peroxide have additive antibacterial activity in combination

**Figure 6:**
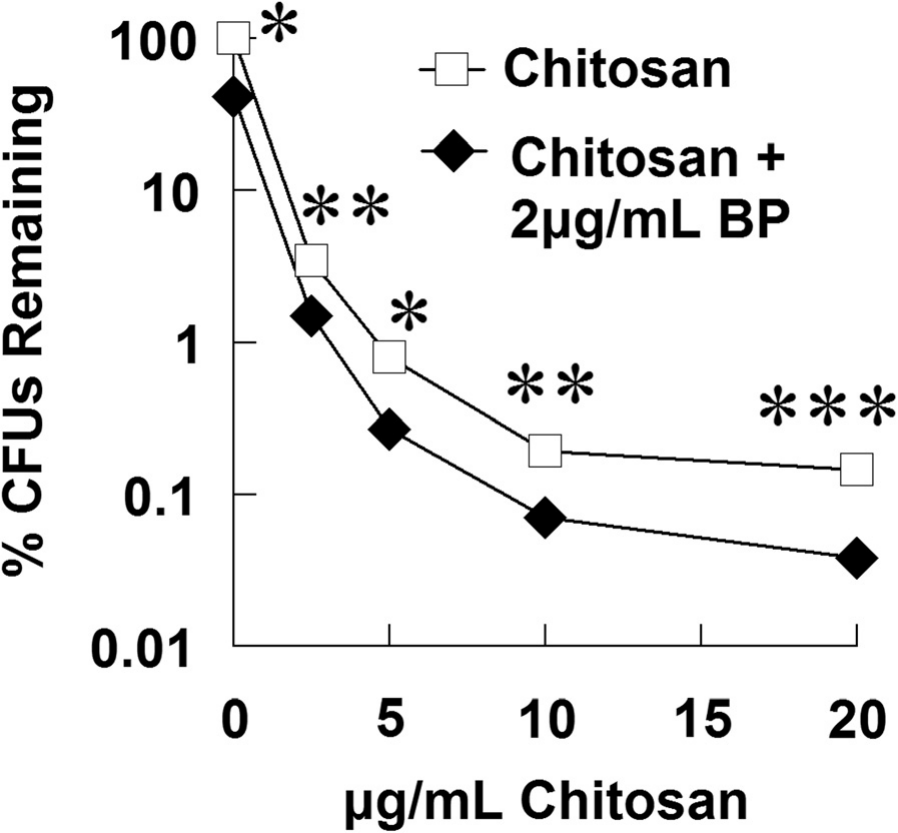
**Chitosan and benzoyl peroxide have additive antibacterial activity in combination.** Chitosan was incubated with *P. acnes* in the presence or absence of benzoyl peroxide for 3 hours to assess antibacterial activity. Bacteria was then enumerated by colony-forming unit assay to determine concentration, which was plotted as a percentage of untreated sample. Error bars represent SEM (n = 3, independent experiments). *p < 0.05, **p < 0.01, and ***p < 0.001 (paired t-test).

Benzoyl peroxide (BP), a common treatment for acne vulgaris, was tested in combination with chitosan against *P. acnes* (Figure 6). Amine groups, particular tertiary amines, are thought to catalyze the antimicrobial mechanism of benzoyl peroxide (Burkhart and Burkhart 2007), and chitosan has a primary amine group. We found that BP at 2 μg/mL inhibited *P. acnes* growth by a half log. Together, BP and chitosan had additive but not synergistic antimicrobial activity against *P. acnes*.

### Chitosan is less toxic to human cells than benzoyl peroxide

**Figure 7:**
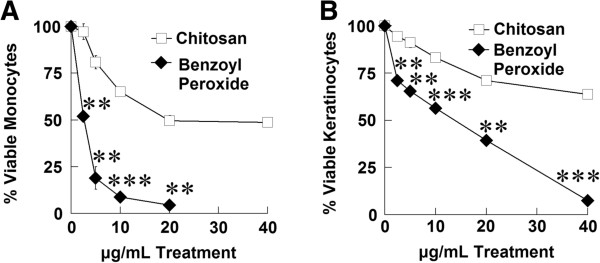
**Chitosan is less toxic to human cells than benzoyl peroxide.** Human monocytes **(A)** or keratinocytes **(B)** were incubated with chitosan or benzoyl peroxide for 16 hours. Cell viability was then determined by MTS assay. Error bars represent SEM **(**n = 3, independent experiments for **A**, one experiment for **B)**. **p < 0.01, and ***p < 0.001 (t-test).

One major adverse effect of benzoyl peroxide as a treatment for acne vulgaris is its high toxicity to human cells. Clinically, BP leads to irritation, especially at a higher concentrations, decreasing patient compliance. We compared the cytotoxicity of chitosan with BP using a 3-(4,5-dimethylthiazol-2-yl)-5-(3-carboxymethoxyphenyl)-2-(4-sulfophenyl)-2H-tetrazolium (MTS) assay. We found that that chitosan is significantly less toxic to keratinocytes and primary human monocytes than benzoyl peroxide (Figure 7). Furthermore, BP concentrations of 20–40 μg/mL results in nearly 100% cell death, but many cells survive when exposed to similar concentrations of chitosan.

## Discussion

As bacteria develop resistance to common antibiotic therapies, there is a need to develop new treatment options. This public health issue is clearly evident in acne vulgaris, which affects over 80% of the population at some point in their lives. Therefore, we assessed the potential of chitosan as an antimicrobial agent against two common cutaneous pathogens. In addition, we gathered data on the antibacterial mechanism of chitosan, and determined how certain properties of chitosan affect its antibacterial activity.

Chitosan appears to only be effective against bacteria when dissolved. Due to its pKa of ~6.3, chitosan is only weakly soluble at neutral pH. We found that chitosan had little antimicrobial activity above pH 7, so it was necessary to keep chitosan at sufficiently low pH to enhance its antimicrobial effect. It is anticipated that chitosan is most effective at lower pH when more of the amine groups are positively charged. Chitosan could then bind to negatively charged bacterial surface, facilitating its antibacterial activity.

By comparing two very different cutaneous pathogens, we found other factors affect chitosan’s antimicrobial effectiveness. *S. aureus* multiplied more quickly when closest to pH 7 in our experiments. At the same time, we measured increased chitosan effect against these more rapidly growing bacteria, as long as the chitosan remained at least partially dissolved. *P. acnes* did not multiply during chitosan treatment, and we found little effect of pH on antimicrobial activity for dissolved chitosan. Additionally, chitosan activity was significantly reduced against both bacterial species in buffers that did not include 0.03% Trypticase Soy Media or other nutrients, despite the fact that this media completely precipitates and inactivates chitosan at higher concentrations. This could potentially be due to a change in bacterial surface characteristics in response to an environment devoid of nutrients, hastening transition to a static growth phase. Moreover, we observed reduced effectiveness of chitosan with increasing NaCl concentration, which also slowed *S. aureus* growth (unpublished data).

Bacterial surface characteristics may account for chitosan’s improved antimicrobial activity against rapidly growing bacteria at higher pHs. One study correlated surface hydrophobicity with chitosan binding (Chung and Su, 2004). *Escherichia coli* in the exponential phase of growth have greater negative surface charge and decreased hydrophobicity (Walker et al. 2005), and other species of bacteria may follow this pattern. This would allow chitosan to have higher affinity for bacteria in the exponential phase, and thus, greater activity against them. However, one study found surface charge and hydrophobicity largely unchanged between the mid-exponential and stationary phases in *S. aureus* (Beck et al. 1988), while another found variable increase or decrease in hydrophobicity between the mid-exponential and stationary phases depending on strain (Baselga et al. 1992). Therefore, other factors may be involved, such as increased protonation of surface proteins reducing chitosan binding at low pH.

Our electron microscopy examination *P. acnes* under chitosan treatment was similar to previous results on gram-positive bacteria (Moon et al. 2007; Raafat et al. 2008; Eaton et al. 2008; Fernandes et al. 2009). We saw that chitosan adheres to the surface of the bacteria cell wall, a conclusion supported by our fluorescence microscopy study. Additionally, the osmotic pressure-induced disruption and shrinkage of the bacterial membrane was consistent with a proposed antibacterial mechanism involving a reduction in the permeability of the membrane to intracellular components (Young et al. 1982). Indeed, other studies have shown potassium leakage (Raafat et al. 2008) as well as protein and nucleotide leakage (Chung and Chen 2008) in chitosan-treated bacteria. This mechanism is bactericidal, as opposed to the bacteriostatic mechanism of several common antibiotics used to treat acne, possibly giving chitosan treatment an advantage.

Another proposed antibacterial mechanism involves chitosan forming a barrier on the bacterial surface, preventing entrance of nutrients (Zheng and Zhu 2003). Higher molecular weight chitosan would be more effetive in this role, which is consistent with our results and previous results, though only in gram-positive bacteria (Tayel et al. 2010). Also in support of this mechanism, we observed bacteria specimens covered with chitosan via electron microscopy, and bacteria in large aggregates of chitosan via florescence microscopy. It is possible that chitosan operates against bacteria via multiple mechanisms.

None of our 8 clinical isolates demonstrated resistance to chitosan, which has a physical attack mechanism, rather than affecting the bacterial machinery like conventional antibiotics. Because of this, any developed resistance to chitosan would most likely be nonspecific, such as a reduction in surface charge or increase in surface hydrophobicity. These changes may reduce bacterial virulence. Additionally, we have shown that chitosan can be combined with benzoyl peroxide, which is capable of destroying drug-resistant bacteria, but irritating. A combined treatment could avoid side effects associated with highly concentrated benzoyl peroxide, as has been done previously with other drugs (Zeichner 2012; Taylor and Shalita 2004).

Major symptoms of acne vulgaris include inflammation and irritation. To counter these effects, a treatment for acne vulgaris should be anti-inflammatory. Our preliminary data suggests that *in vitro*, chitosan does not reduce induction of inflammatory cytokines in *P. acnes*-stimulated monocytes (unpublished data). We have yet to test the effect of chitosan in other pathways of inflammation involved in the pathogenesis of acne, including the regulation of inflammatory lipids. Indeed, chitosan has been shown to affect lipid binding (Wydro et al. 2007) and is currently used as diet supplement. Furthermore, chitosan has been shown to increase the permeability of epithelial cell junctions (Smith et al 2004). This could allow treatments with chitosan to reach deeper into pilosebaceous follicles or dermal layers. Further studies are warranted to test these properties in acne and other models of skin infection.

Overall, chitosan has been shown to be effective against *P. acnes* and *S. aureus* under specific conditions *in vitro*. To be effective clinically, chitosan activity in biological environments must be studied. Indeed chemical modification of chitosan has shown some promising data (Chen and Chou 2005; Mohy Eldin et al. 2008; Ji et al. 2009). Nevertheless, this is the first study to demonstrate that chitosan has several properties that make it a very promising choice for effective treatment of *P. acnes* infection. Future studies are needed to develop an effective way to use chitosan *in vivo*.

## Competing interests

The authors declare that they have no competing interest.
